# Role of Leptin in Mood Disorder and Neurodegenerative Disease

**DOI:** 10.3389/fnins.2019.00378

**Published:** 2019-05-03

**Authors:** Xiaohan Zou, Lili Zhong, Cuilin Zhu, Haisheng Zhao, Fangyi Zhao, Ranji Cui, Shuohui Gao, Bingjin Li

**Affiliations:** ^1^Jilin Provincial Key Laboratory on Molecular and Chemical Genetic, The Second Hospital of Jilin University, Changchun, China; ^2^Department of Gastrointestinal Colorectal Surgery, China-Japan Union Hospital of Jilin University, Changchun, China

**Keywords:** leptin, neurodegeneration, mood disorders, depression, neuroprotection

## Abstract

The critical regulatory role of leptin in the neuroendocrine system has been widely reported. Significantly, leptin can improve learning and memory, affect hippocampal synaptic plasticity, exert neuroprotective efficacy and reduce the risk of several neuropsychiatric diseases. In terms of depression, leptin could modulate the levels of neurotransmitters, neurotrophic factors and reverse the dysfunction in the hypothalamic-pituitary-adrenal axis (HPA). At the same time, leptin affects neurological diseases during the regulation of metabolic homeostasis. With regards to neurodegenerative diseases, leptin can affect them via neuroprotection, mainly including Alzheimer’s disease and Parkinson’s disease. This review will summarize the mechanisms of leptin signaling within the neuroendocrine system with respect to these diseases and discuss the therapeutic potential of leptin.

## Introduction

Leptin is an adipocyte-derived hormone which is encoded by the obese gene (Zhang et al., 1994). Receptors of leptin are expressed in many brain regions, such as the arcuate nucleus of the hypothalamus, olfactory bulb, the dorsal raphe nucleus, hippocampus, the cortex and the nucleus of the solitary tract (Tartaglia et al., 1995). Recently, growing experimental results indicate that leptin also plays a significant regulatory role in the central nervous system (CNS) and is associated with several pathological and physiological mechanisms of neurological diseases, including neurodegenerative diseases and mood disorders (Lee et al., 2015; Kurosawa et al., 2016). It was found that neurological diseases occurred alongside leptin level alterations, indicating that leptin might be a critical modulator of these diseases and studying the specific relationship is of significance. In this article, we mainly discuss the role of leptin in mood disorder and neurodegenerative diseases and try to interpret the potential mechanisms.

## The Role of Leptin in Depression

Depression is one of the most prevalent mental illnesses, with high morbidity and suicide rates (Milaneschi et al., 2017). Due to the serious side-effects and long onset time of traditional antidepressants, recent investigations focus on neuropeptides’ antidepressant effects and potential mechanisms, such as leptin and ghrelin (Kormos and Gaszner, 2013). Clinical studies investigating the relationship of depression and leptin levels yielded inconsistent results. Lower leptin levels were reported in depressive patients compared to controls in earlier studies. However, there is also research demonstrating that patients with major depression disorder have higher leptin levels (Milaneschi et al., 2017). The confounding factors, including age, gender, and medication history of depressive patients, might impact periphery leptin levels (Ge et al., 2018). Several animal studies demonstrated lower leptin levels in rats with chronic unpredictable stress (Ozsoy et al., 2015). Pharmacological studies have shown that intra-hippocampus administration of leptin could exert an antidepressant-like effect, while no positive efficacy has been detected when leptin was injected into the hypothalamus (Finn et al., 2001; Lu et al., 2006). Leptin can also increase the activation of neurons in hippocampal limbic structures which contribute to a delayed long-lasting antidepressant-like effect in force swim test (Kurosawa et al., 2016). Deletion of leptin receptor (LepRb) is sufficient to induce depression-like behavioral impairments, indicating that leptin-lepRb signaling is involved in the molecular mechanism of leptin’s antidepressant action (Guo et al., 2013). However, the possible molecular and cellular mechanisms of leptin’s antidepressant actions are still obscure.

## Leptin’s Role in Neurotransmission

Both basic and clinical investigations demonstrate that the brains of patients with depression are characterized by disturbances of the neurotransmitter system, including 5-hydroxytryptamine (5-HT), dopamine (DA) and γ-aminobutyric acid (GABA). Traditional depression theories propose that a lack of 5-HT leads to depression, and monoaminergic drugs can alleviate behavior impairments (Aberg-Wistedt et al., 1998). It was reported that leptin administration decreases the binding site density of the selective 5-HT transporter inhibitor paroxetine (Aberg-Wistedt et al., 1998; Charnay et al., 2000). The 5-HT transporter mRNA levels are lower in leptin-deficient ob/ob mice (Collin et al., 2000). These results suggest that leptin can promote the 5-HT transporter functionally and enhance the expression in protein levels. DA has the potential to be an antidepressant drug (Jay et al., 2004). Double-labeling fluorescence immunohistochemistry suggests that dopamine neurons also express leptin receptors in the brain (Figlewicz et al., 2003). Leptin can impact motivated behavior and reward-seeking behavior via the midbrain DA pathway (Fulton et al., 2006). In addition, the level of GABA in depressed patients is lower than that in healthy subjects (Sanacora et al., 1999). Antidepressants drugs can alleviate depressive phenotypes via activation of the GABA transmission system (Garcia-Garcia et al., 2009; Fuchs et al., 2017). As there is expression of LepRb on GABAergic neurons, leptin potentially exerts regulatory effects via the GABAergic system (Fuchs et al., 1984; Francis et al., 2004).

## Leptin’s Neurotrophic Effect

The current neurotrophic hypothesis of depression proposed that a deficit of neurotrophic factors or disturbance of neurotrophic factor signaling pathways is the primary cause of depression (Gulyaeva, 2017). Brain-derived neurotrophic factor (BDNF) is a member of the neurotrophin protein family and is involved in the pathophysiological symptoms of depression (Novkovic et al., 2015; Huang et al., 2017). BDNF could influence hippocampal synaptic plasticity through down-regulating 5-HT3 receptors (Hao et al., 2017).

Leptin was reported to increase the expression of BDNF mRNA (Komori et al., 2006). Leptin can also activate BDNF-expressing hypothalamic neurons through activating neural circuits that stimulate dendritic BDNF synthesis (Liao et al., 2012). BDNF plays a key role in the CNS through binding its receptor. Administration of leptin to the hindbrain significantly increases the level of BDNF within the dorsal vagal complex (Sahu et al., 2016; Kim et al., 2017).

Leptin can significantly improve cAMP-response element binding protein (CREB) phosphorylation via the MAP kinase/extracellular signal-regulated protein kinase (ERK1/2) pathway (Dhar et al., 2014). ERK1/2 phosphorylation (pERK1/2) can directly activate the protein signaling cascade to regulate a series of cellular processes, such as nerve growth, survival and neuroplasticity. Leptin can induce ERK1/2 phosphorylation in a time-dependent manner (Kim et al., 2017; Ghasemi et al., 2018; Han et al., 2018). The increase in pERK1/2 can phosphorylate CREB and alter its transcriptional activity, which is considered a key event of cell survival and cognition (Liu et al., 2015) and in the case of cocultured neurons and astrocytes, leptin exerts an anti-apoptotic effect in astrocytes against glutamate toxicity (Park et al., 2017).

The BDNF and phosphatidylinositol 3 kinase (PI3K)/protein kinase B (AKT) pathways not only regulate the growth and survival of neurons in the hippocampus, but also regulate stress-induced depression and antidepressant response. Several recent studies have found that the antidepressant effect of antidepressants may be related to the PI3K-AKT-mammalian target of rapamycin (mTOR) pathway. Treatment with leptin activates the PI3K-AKT-mTOR pathway (Fazolini et al., 2015; Gui et al., 2018). BDNF increased outgrowth of hippocampal neurites though PI3K pathway signaling (Park et al., 2013). Administration of exogenous leptin to SD rats induced up-regulation of Janus Kinase 2 (JAK2)-signal transducers, and activators of transcription 3 (STAT3) signaling (Wu et al., 2017). To summarize, the protein levels of pSTAT3, AKT, and ERK are all up-regulated by leptin (Kim et al., 2017).

## Leptin and Hypothalamic-Pituitary-Adrenal Axis

Elevation of hypothalamic-pituitary-adrenal axis (HPA) activity is one of the most common neurobiological abnormalities in patients with depression. Studies have shown that the most important factor in the increase of hypothalamic-pituitary activity is the excessive secretion of corticotropin-releasing hormone (CRH) (Plotsky et al., 1998; Morris et al., 2012). CRH induces pituitary adrenal corticotropic hormone (ACTH) secretion; in turn, ACTH causes the adrenal cortex to secrete glucocorticoids (GC). When the concentration of GC increases (e.g., during stress), GC binds to the glucocorticoid receptor (GR), causing negative feedback to inhibit CRH in the hypothalamus. Finally, the hyperactive HPA axis is restored to the level at baseline (Juruena, 2014). However, hypersecretion of GC constantly stimulates GR, leading to GR desensitization (Board et al., 1957; Cowen, 2010).

Leptin leads to the down-regulation of CRH in the paraventricular hypothalamic nucleus (PVH), and small doses of leptin can also down-regulate CRH mRNA expression. This function of leptin demonstrates that it is a regulator of the HPA axis (Arvaniti et al., 2001). In a starvation model leptin is used to change HPA axis activity. Leptin prevents the synthesis of CRH in PVN and inhibits the activation of the CRH neurons (Huang et al., 1998). Plasma leptin inhibits the expression of the ACTH receptor (ACTH-R) (Su et al., 2012). Furthermore, an injection of leptin to the sheep fetus inhibits the rise in ACTH and cortisol concentration (Howe et al., 2002). Besides the known effects of leptin on ACTH, ACTH can modulate leptin secretion in plasma. Increased plasma ACTH concentrations cause a decrease in leptin output (Spinedi and Gaillard, 1998).

## Leptin and Metabolic Abnormalities in Neurological Diseases

Metabolic homeostasis is a complicated regulation process that implicates regulatory signals from both CNS and peripheral systems (Procaccini et al., 2016). As leptin is an important peripheral signal molecule, it is necessary to take metabolic factors into account. Leptin resistance, manifesting as feedback elevated peripheral levels, is defined as a hallmark of metabolic disorders (Talton et al., 2016; Szkudelski et al., 2017; Wang et al., 2018). Recent studies gave the explanation that leptin resistance is caused by leptin signaling disruption, which implicates LepRb deficiency, leptin transport dysfunction through the blood–brain barrier (BBB) and intracellular leptin signaling pathways defects (Wang et al., 2014). Obesity is the most prevalent side-effect of present therapeutic drugs for neurological diseases (Maayan and Correll, 2010). Long-lasting metabolic abnormalities lead to leptin resistance and leptin signaling disruption (Pan et al., 2014). In turn, epidemiological studies showed that diabetes patients have an increased risk of depression and Alzheimer’s disease (AD) compared to people without diabetes (Anderson et al., 2001; Arvanitakis et al., 2004; Ernst et al., 2013). These results suggest that neurological diseases, especially mood disorders and metabolism abnormalities, might share overlapping brain circuitries integrating homeostatic and mood regulatory responses and genetic susceptibility factors. As a neuroendocrine regulator of energy metabolism, circulating leptin levels appear to change immediately, which is correlated with central leptin signaling disruption.

Ottaway et al. (2015) found that obese animals retain their sensitivity to endogenous leptin; however, that does not argue against the presence of leptin resistance, based on the most recent reports. For instance, there are increases in plasma leptin concentrations during the initial stage of pregnancy, down-regulation of hypothalamic long form of the leptin receptor in the ventro- and dorso-medial nuclei during the second half of gestation and suppressor of cytokine signaling-3 up-regulation in the arcuate nucleus in late-pregnant ewes (Szczesna et al., 2019). In studying age-related obesity, celastrol, a leptin sensitizer, can induce weight loss in aged animals but not in young controls (Chellappa et al., 2019). In addition, gene expression of leptin receptor in the hypothalamus was found significantly down-regulated in a high-fat diet group (Zhao et al., 2018). These findings support the presence of a relative “leptin resistance” despite partial activity of endogenous leptin signaling in obese animals.

In addition, several experiments *in vivo* and *in vitro* confirmed that leptin itself could exert neuroprotective and neurotrophic actions via promoting BDNF signaling and reduction of neuronal apoptotic and loss (Spina et al., 1992; Komori et al., 2006; Novkovic et al., 2015). These might explain why leptin can improve cognitive and behavior impairments. Contradictory observations exist showing that fasting and calorie restriction, contributing to a decreased leptin level, have an anti-depressant effect (Alzoghaibi et al., 2014; Zhang et al., 2015). Since most animal studies use a few hours of fasting as an experimental process, leptin’s antidepressant action is a comparably long-term process. It can be inferred that they may exert antidepressant actions via different molecular ways, while the clear mechanisms are still obscure. In conclusion, leptin might be a potential combination therapeutic target but still not sensitive enough to be a biomarker of neurological diseases at present.

## Leptin’s Neuroprotective Effect in Neurodegenerative Disease

### Leptin and Alzheimer’s Disease

Alzheimer’s disease is one of the most common chronic neurodegenerative diseases and mainly occurs in the elderly (Mangialasche et al., 2010). Amyloid-β, neurofibrillary tangles, synaptic loss and reactive gliosis are the major neuropathological hallmarks of AD (Rockenstein et al., 1995; Alpár et al., 2006).

Previous studies demonstrated that neurotrophic and neuroprotective effects have been induced by leptin in Alzheimer’s patients (Pérez-González et al., 2011). Amyloid-β, the main component of amyloid plaques, is highly expressed in the brains of AD patients. It has been observed that the amyloid-β level is decreased in both brain extracts and the serum of transgenic mice after treatment with leptin (Xing et al., 2015). Immunocytochemistry analysis also revealed a decrease in amyloid-β levels in the hippocampus (Greco et al., 2010; Xing et al., 2015). The phosphorylation of JAK2, STAT3 and the consequent activation of adenosine 5′-monophosphate (AMP)-activated protein kinase (AMPK) are involved, whereas it has also been found that primary neurons exhibit increased amyloid-β levels following leptin antagonist treatment (Liu et al., 2017). As showed in Table 1, several animal studies reported leptin have significant regulatory role in AD and depression. Leptin phosphorylates PI3K/AKT/mTOR to decrease the expression of GM1 ganglioside in the detergent-resistant membrane microdomains (DRMs) of the neuronal surface. Subsequently, the decrease of GM1 ganglioside (GM1) inhibits the assembly of amyloid-β (Yamamoto et al., 2014). In addition, outgrowth of neurites in primary neuronal cultures is influenced by leptin. Leptin can rescue the neurite from amyloid-β toxicity (Pérez-González et al., 2014). Chronic leptin treatment is able to recover the deficits caused by amyloid-β. Leptin rescues deficits in spatial memory induced by amyloid-β and long-term potentiation *in vivo* in the hippocampal late-phase. Chronic intracerebroventricular injection of leptin alleviates spatial memory impairment (Tong et al., 2015). Administration of leptin also reverses amyloid-β-induced suppression of hippocampal late-phase long-term potentiation in rats (Tong et al., 2015).

**Table 1:** Role of leptin in neurological diseases.

Disease	References	Model	Role of leptin
AD	Dudek and Bear, 1992; Mulkey and Malenka, 1992	Long-term potentiation and high-frequency stimulation in hippocampal synapses	Enhances NMDA receptor
AD	Dicou et al., 2001	Ibotenate increase cortical lesions and white matter cysts	Activates its receptor and JAK2
AD	Yamamoto et al., 2014	GM1 ganglioside in the detergent-resistant membrane microdomains (DRMs) of neuronal surface	Decreases GM1 and inhibits the assembly of amyloid-β
Depression	Kurosawa et al., 2016	Forced swim test	Increases the activation of neurons in hippocampus limbic structures
Depression	Park et al., 2017	Coculture neurons and astrocytes	Exerts an anti-apoptotic effect in astrocytes, acting against glutamate

Leptin can affect hippocampus-dependent learning and memory processes (Kiliaan et al., 2014). With regards to long-term potentiation and high-frequency stimulation in hippocampal synapses, synaptic activation of *N*-methyl-D-aspartate (NMDA) receptors is important (Dudek and Bear, 1992; Mulkey and Malenka, 1992). Leptin affects hippocampal synaptic plasticity by enhancing the expression of NMDA receptors (Kiliaan et al., 2014). It has also been shown that AβPP/PS1 double transgenic mice, a mouse model for AD, display increased caspase-3 expression and a reduction in synapse number, which can be reversed to the previous state by leptin treatment (Pérez-González et al., 2014). At the same time, leptin can reduce cortical lesions and white matter cysts. Results from *in vitro* experiments showed that leptin might act as a potential neuroprotective factor. Activation of the leptin receptor and consequent JAK2 are involved in this process (Dicou et al., 2001). In addition, leptin can stimulate neuronal proliferation. It has been reported that chronic leptin administration increases BrdU-positive cells in the dentate gyrus subgranular zone of the hippocampus which indicates a neurogenesis-stimulated benefit of leptin (Pérez-González et al., 2011).

Microglial cells are classes of immune cells that modulate homeostasis in the brain. In the brain of patients with AD, the level of microglia clearance tends to be insufficient (Bacskai et al., 2001; Napoli and Neumann, 2009). On the other hand, some studies have suggested that phagocytosis of microglia leads to the death of neurons. Lipoteichoic acid and lipopolysaccharide (agonists of glial TLR2 and TLR4, respectively) also activate microglia phagocytes, leading to inflammatory neurodegeneration (Neher et al., 2011). It has been shown that leptin deficiency or leptin antagonists inhibit the development of microgliosis in the brain. Thus, leptin is involved in the proliferation of microglia (Fernández-Martos et al., 2012; Gao et al., 2014; Chang et al., 2017). However, the association of leptin’s effect on microglia and development of AD needs further exploration.

Several animal studies have confirmed leptin’s effect on AD, such as its neurotrophic and neuroprotective effects, its decreasing amyloid-β level, its rescuing the neurites from amyloid-βtoxicity, its influencing hippocampus-dependent learning and memory processes and so on. However, some results from human studies have shown that plasma leptin level has no effect on cognitive ability. It has therefore been suggested that plasma leptin is not an appropriate clinical biomarker for AD at this stage (Oania and McEvoy, 2015; Teunissen et al., 2015).

### Leptin and Parkinson’s Disease

Parkinson’s disease, another common neurodegenerative disease, is characterized by classical motor function deficits due to loss of dopaminergic neurons in the substantia nigra and is induced by a complicated interplay between genetic and environmental factors (Kalia and Lang, 2015).

It was well-known that Parkinson’s disease (PD) was mainly characterized by death of dopaminergic neurons in substantia nigra and the accumulation of proteins into Lewy bodies in the neurons (Cosgrove et al., 2015; Duda et al., 2016). Studies of 6-hydroxydopamine (6-OHDA)-induced PD animal models showed that leptin can reverse behavioral abnormalities and reduced dopaminergic cell death (Weng et al., 2007). In the process of leptin-induced neuroprotection, extracellular regulated pERK1/2 plays a key role as a survival factor of dopaminergic neurons, which caused subsequently a MEK-dependent increase in CREB (Weng et al., 2007). Furthermore, another downstream product of leptin is BDNF, which can preserve the survival of dopaminergic neurons via activation of the ERK/CREB pathway (Spina et al., 1992). Though some human studies showed that there’s no significant correlation of peripheral leptin levels and PD, it was found that circulating leptin levels of unintended weight loss PD patients were lower than those with stable weight (Evidente et al., 2001; Fiszer et al., 2010). Different selection criteria for inclusion might explain the contradictory conclusions.

Leptin can also preserve neuronal survival via increased uncoupling protein-2 (UCP2) expression in neuronal cultures. UCP2 could maintain the level of ATP and mitochondrial membrane potential (MMP). At the same time, it preserves cell survival against MPP+ toxicity, which has been widely used in producing Parkinsonism models (Ho et al., 2010; Procaccini et al., 2016). These results suggest that leptin might have potential to be a therapeutic target. However, at this stage, the research is relatively limited. More research will be needed to address this issue.

### The Therapeutic Potential of Leptin

In the context of increasing incidence of neurological diseases, it is important to explore the pathogenesis of these diseases and to find effective treatments. It has been shown that leptin has an effect on the nervous system. Leptin could modulate the levels of neurotransmitters, promote the 5-HT transporter functionally and enhance the expression in protein levels (Collin et al., 2000). Also, there is expression of LepRb on GABAergic neurons and dopamine neurons in the brain (Fuchs et al., 1984; Figlewicz et al., 2003; Francis et al., 2004). Leptin can also increase the expression of BDNF mRNA, activate BDNF-expressing neurons (Komori et al., 2006; Liao et al., 2012), activate the PI3K-AKT-mTOR pathway to regulate the growth of neurons and regulate stress-induced depression and antidepressant response (Fazolini et al., 2015; Gui et al., 2018) while reversing the dysfunction in the HPA axis. These functions of leptin reflect its potential to treat depression. In neurodegenerative disease, leptin has neurotrophic and neuroprotective effects (Pérez-González et al., 2011), it affects hippocampal synaptic plasticity and improves learning and memory processes (Kiliaan et al., 2014).

However, some experiments from human studies have shown that plasma leptin levels are not associated with these diseases. Studies have shown that leptin levels are higher in depression patients than in control groups (Milaneschi et al., 2017). Moreover, leptin has no effect on human cognition and memory ability (Oania and McEvoy, 2015; Teunissen et al., 2015). Thus, despite the fact that leptin has the potential to be a therapeutic drug for neurological diseases through different molecular mechanisms and a target for combination therapy, it is not a clinical biomarker for neurological diseases before a clear mechanism is explored.

## Conclusion

Since the prevalence of neurodegenerative disorders and mood disorders has ascended in recent years, investigating the radical cellular and molecular mechanisms of these diseases and finding out a novel therapeutic target is important. In this article, we discussed the effects of adipocyte-derived hormone leptin in depression, AD, PD and its possible modulatory role. Antidepressant effects of leptin have been observed in recent studies. The mechanism might implicate leptin’s role in neurotransmission, neurotrophic factors and the HPA axis. Furthermore, an inescapable issue is that neurological diseases and metabolism abnormalities might share overlapping brain circuitries integrating homeostatic and regulatory responses and genetic susceptibility factors. Still, increasing evidence indicates a potential effect of leptin in reversing AD symptoms. The effect of leptin might be based on the mechanism that increases the activation of neurons in the hippocampus, reduces the levels of amyloid-β and tau and modulates the microglia. As for PD, leptin can preserve dopaminergic neurons via several pathways. Leptin appears to exert neuroprotective effects on neurodegenerative disorders. More investigation is required to understand the association between leptin and neurological diseases.

## Author Contributions

XZ, LZ, and CZ wrote the first draft. XZ, LZ, CZ, HZ, FZ, RC, SG, and BL participated in the discussion of the manuscript. SG and BL provided the critical revisions. All authors approved the final version of the manuscript for submission.

## Conflict of Interest Statement

The authors declare that the research was conducted in the absence of any commercial or financial relationships that could be construed as a potential conflict of interest.

## References

[B1] Aberg-Wistedt A. Hasselmark L. Stain-Malmgren R. Apéria B. Kjellman B. F. Mathé A. A. (1998). Serotonergic ‘vulnerability’ in affective disorder: a study of the tryptophan depletion test and relationships between peripheral and central serotonin indexes in citalopram-responders. *Acta Psychiatr. Scand.* 97 374–380. 9611088 10.1111/j.1600-0447.1998.tb10017.x

[B2] Alpár A. Ueberham U. Brückner M. K. Seeger G. Arendt T. Gärtner U. (2006). Different dendrite and dendritic spine alterations in basal and apical arbors in mutant human amyloid precursor protein transgenic mice. *Brain Res.* 1099 189–198. 10.1016/j.brainres.2006.04.109 16781686

[B3] Alzoghaibi M. A. Pandi-Perumal S. R. Sharif M. M. BaHammam A. S. (2014). Diurnal intermittent fasting during Ramadan: the effects on leptin and ghrelin levels. *PLoS One* 9:e92214. 10.1371/journal.pone.0092214 24637892 PMC3956913

[B4] Anderson R. J. Freedland K. E. Clouse R. E. Lustman P. J. (2001). The prevalence of comorbid depression in adults with diabetes: a meta-analysis. *Diabetes Care* 24 1069–1078. 11375373 10.2337/diacare.24.6.1069

[B5] Arvanitakis Z. Wilson R. S. Bienias J. L. Evans D. A. Bennett D. A. (2004). Diabetes mellitus and risk of Alzheimer disease and decline in cognitive function. *Arch. Neurol.* 61 661–666. 10.1001/archneur.61.5.661 15148141

[B6] Arvaniti K. Huang Q. Richard D. (2001). Effects of leptin and corticosterone on the expression of corticotropin-releasing hormone, agouti-related protein, and proopiomelanocortin in the brain of ob/ob mouse. *Neuroendocrinology* 73 227–236. 10.1159/000054639 11340336

[B7] Bacskai B. J. Kajdasz S. T. Christie R. H. Carter C. Games D. Seubert P. (2001). Imaging of amyloid-beta deposits in brains of living mice permits direct observation of clearance of plaques with immunotherapy. *Nat. Med.* 7 369–372. 10.1038/85525 11231639

[B8] Board F. Wadeson R. Persky H. (1957). Depressive affect and endocrine functions; blood levels of adrenal cortex and thyroid hormones in patients suffering from depressive reactions. *AMA Arch. Neurol. Psychiatry* 78 612–620. 13478217

[B9] Chang K. T. Lin Y. L. Lin C. T. Hong C. J. Tsai M. J. Huang W. C. (2017). Leptin is essential for microglial activation and neuropathic pain after preganglionic cervical root avulsion. *Life Sci.* 187 31–41. 10.1016/j.lfs.2017.08.016 28822786

[B10] Charnay Y. Cusin I. Vallet P. G. Muzzin P. Rohner-Jeanrenaud F. Bouras C. (2000). Intracerebroventricular infusion of leptin decreases serotonin transporter binding sites in the frontal cortex of the rat. *Neurosci. Lett.* 283 89–92. 10739882 10.1016/s0304-3940(00)00951-4

[B11] Chellappa K. Perron I. J. Naidoo N. Baur J. A. (2019). The leptin sensitizer celastrol reduces age-associated obesity and modulates behavioral rhythms. *Aging Cell* 10.1111/acel.12874 [Epub ahead of print]. 30821426 PMC6516176

[B12] Collin M. Håkansson-Ovesjö M. L. Misane I. Ogren S. O. Meister B. (2000). Decreased 5-HT transporter mRNA in neurons of the dorsal raphe nucleus and behavioral depression in the obese leptin-deficient ob/ob mouse. *Brain Res. Mol. Brain Res.* 81 51–61. 11000478 10.1016/s0169-328x(00)00167-4

[B13] Cosgrove J. Alty J. E. Jamieson S. (2015). Cognitive impairment in Parkinson’s disease. *Postgrad. Med. J.* 91 212–220. 10.1136/postgradmedj-2015-133247 25814509

[B14] Cowen P. J. (2010). Not fade away: the HPA axis and depression. *Psychol. Med.* 40 1–4. 10.1017/S0033291709005558 19335939

[B15] Dhar M. Zhu M. Impey S. Lambert T. J. Bland T. Karatsoreos I. N. (2014). Leptin induces hippocampal synaptogenesis via CREB-regulated microRNA-132 suppression of p250GAP. *Mol. Endocrinol.* 28 1073–1087. 10.1210/me.2013-1332 24877561 PMC4075163

[B16] Dicou E. Attoub S. Gressens P. (2001). Neuroprotective effects of leptin in vivo and in vitro. *Neuroreport* 12 3947–3951. 11742217 10.1097/00001756-200112210-00019

[B17] Duda J. Pötschke C. Liss B. (2016). Converging roles of ion channels, calcium, metabolic stress, and activity pattern of Substantia nigra dopaminergic neurons in health and Parkinson’s disease. *J. Neurochem.* 139(Suppl. 1) 156–178. 10.1111/jnc.13572 26865375 PMC5095868

[B18] Dudek S. M. Bear M. F. (1992). Homosynaptic long-term depression in area CA1 of hippocampus and effects of N-methyl-D-aspartate receptor blockade. *Proc. Natl. Acad. Sci. U.S.A.* 89 4363–4367. 1350090 10.1073/pnas.89.10.4363 PMC49082

[B19] Ernst A. Sharma A. N. Elased K. M. Guest P. C. Rahmoune H. Bahn S. (2013). Diabetic db/db mice exhibit central nervous system and peripheral molecular alterations as seen in neurological disorders. *Transl. Psychiatry* 3:e263. 10.1038/tp.2013.42 23715298 PMC3669927

[B20] Evidente V. G. Caviness J. N. Adler C. H. Gwinn-Hardy K. A. Pratley R. E. (2001). Serum leptin concentrations and satiety in Parkinson’s disease patients with and without weight loss. *Mov. Disord.* 16 924–927. 11746624 10.1002/mds.1165

[B21] Fazolini N. P. Cruz A. L. Werneck M. B. Viola J. P. Maya-Monteiro C. M. Bozza P. T. (2015). Leptin activation of mTOR pathway in intestinal epithelial cell triggers lipid droplet formation, cytokine production and increased cell proliferation. *Cell Cycle* 14 2667–2676. 10.1080/15384101.2015.1041684 26017929 PMC4614828

[B22] Fernández-Martos C. M. González P. Rodriguez F. J. (2012). Acute leptin treatment enhances functional recovery after spinal cord injury. *PLoS One* 7:e35594. 10.1371/journal.pone.0035594 22536415 PMC3334982

[B23] Figlewicz D. P. Evans S. B. Murphy J. Hoen M. Baskin D. G. (2003). Expression of receptors for insulin and leptin in the ventral tegmental area/substantia nigra (VTA/SN) of the rat. *Brain Res.* 964 107–115. 12573518 10.1016/s0006-8993(02)04087-8

[B24] Finn P. D. Cunningham M. J. Rickard D. G. Clifton D. K. Steiner R. A. (2001). Serotonergic neurons are targets for leptin in the monkey. *J. Clin. Endocrinol. Metab.* 86 422–426. 10.1210/jcem.86.1.7128 11232034

[B25] Fiszer U. Michałowska M. Baranowska B. Wolińska-Witort E. Jeske W. Jethon M. (2010). Leptin and ghrelin concentrations and weight loss in Parkinson’s disease. *Acta Neurol. Scand.* 121 230–236. 10.1111/j.1600-0404.2009.01185.x 20028343

[B26] Francis J. MohanKumar S. M. MohanKumar P. S. (2004). Leptin inhibits norepinephrine efflux from the hypothalamus in vitro: role of gamma aminobutyric acid. *Brain Res.* 1021 286–291. 10.1016/j.brainres.2004.07.010 15342279

[B27] Fuchs E. Mansky T. Stock K. W. Vijayan E. Wuttke W. (1984). Involvement of catecholamines and glutamate in GABAergic mechanism regulatory to luteinizing hormone and prolactin secretion. *Neuroendocrinology* 38 484–489. 10.1159/000123937 6738812

[B28] Fuchs T. Jefferson S. J. Hooper A. Yee P. H. Maguire J. Luscher B. (2017). Disinhibition of somatostatin-positive GABAergic interneurons results in an anxiolytic and antidepressant-like brain state. *Mol. Psychiatry* 22 920–930. 10.1038/mp.2016.188 27821870 PMC5422144

[B29] Fulton S. Pissios P. Manchon R. P. Stiles L. Frank L. Pothos E. N. (2006). Leptin regulation of the mesoaccumbens dopamine pathway. *Neuron* 51 811–822. 10.1016/j.neuron.2006.09.006 16982425

[B30] Gao Y. Ottaway N. Schriever S. C. Legutko B. García-Cáceres C. de la Fuente E. (2014). Hormones and diet, but not body weight, control hypothalamic microglial activity. *Glia* 62 17–25. 10.1002/glia.22580 24166765 PMC4213950

[B31] Garcia-Garcia A. L. Elizalde N. Matrov D. Harro J. Wojcik S. M. Venzala E. (2009). Increased vulnerability to depressive-like behavior of mice with decreased expression of VGLUT1. *Biol. Psychiatry* 66 275–282. 10.1016/j.biopsych.2009.02.027 19409534

[B32] Ge T. Fan J. Yang W. Cui R. Li B. (2018). Leptin in depression: a potential therapeutic target. *Cell Death Dis.* 9:1096. 10.1038/s41419-018-1129-1 30367065 PMC6203758

[B33] Ghasemi A. Hashemy S. I. Aghaei M. Panjehpour M. (2018). Leptin induces matrix metalloproteinase 7 expression to promote ovarian cancer cell invasion by activating ERK and JNK pathways. *J. Cell. Biochem.* 119 2333–2344. 10.1002/jcb.26396 28885729

[B34] Greco S. J. Bryan K. J. Sarkar S. Zhu X. Smith M. A. Ashford J. W. (2010). Leptin reduces pathology and improves memory in a transgenic mouse model of Alzheimer’s disease. *J. Alzheimers Dis.* 19 1155–1167. 10.3233/JAD-2010-1308 20308782 PMC2862270

[B35] Gui X. Chen H. Cai H. Sun L. Gu L. (2018). Leptin promotes pulmonary fibrosis development by inhibiting autophagy via PI3K/Akt/mTOR pathway. *Biochem. Biophys. Res. Commun.* 498 660–666. 10.1016/j.bbrc.2018.03.039 29524411

[B36] Gulyaeva N. V. (2017). Interplay between brain BDNF and glutamatergic systems: a brief state of the evidence and association with the pathogenesis of depression. *Biochem. Mosc.* 82 301–307. 10.1134/S0006297917030087 28320271

[B37] Guo M. Huang T. Y. Garza J. C. Chua S. C. Lu X. Y. (2013). Selective deletion of leptin receptors in adult hippocampus induces depression-related behaviours. *Int. J. Neuropsychopharmacol.* 16 857–867. 10.1017/S1461145712000703 22932068 PMC3612133

[B38] Han Y. C. Ma B. Guo S. Yang M. Li L. J. Wang S. J. (2018). Leptin regulates disc cartilage endplate degeneration and ossification through activation of the MAPK-ERK signalling pathway in vivo and in vitro. *J. Cell. Mol. Med.* 22 2098–2109. 10.1111/jcmm.13398 29372627 PMC5867127

[B39] Hao R. Qi Y. Hou D. N. Ji Y. Y. Zheng C. Y. Li C. Y. (2017). BDNF val66met polymorphism impairs hippocampal long-term depression by down-regulation of 5-HT3 receptors. *Front. Cell. Neurosci.* 11:306. 10.3389/fncel.2017.00306 29075179 PMC5643500

[B40] Ho P. W. Liu H. F. Ho J. W. Zhang W. Y. Chu A. C. Kwok K. H. (2010). Mitochondrial uncoupling protein-2 (UCP2) mediates leptin protection against MPP+ toxicity in neuronal cells. *Neurotox. Res.* 17 332–343. 10.1007/s12640-009-9109-y 19763737 PMC2946553

[B41] Howe D. C. Gertler A. Challis J. R. (2002). The late gestation increase in circulating ACTH and cortisol in the fetal sheep is suppressed by intracerebroventricular infusion of recombinant ovine leptin. *J. Endocrinol.* 174 259–266. 12176664 10.1677/joe.0.1740259

[B42] Huang Q. Rivest R. Richard D. (1998). Effects of leptin on corticotropin-releasing factor (CRF) synthesis and CRF neuron activation in the paraventricular hypothalamic nucleus of obese (ob/ob) mice. *Endocrinology* 139 1524–1532. 10.1210/endo.139.4.5889 9528930

[B43] Huang X. Huang X. Zhou Y. He H. Mei F. Sun B. (2017). Association of serum BDNF levels with psychotic symptom in chronic patients with treatment-resistant depression in a Chinese Han population. *Psychiatry Res.* 257 279–283. 10.1016/j.psychres.2017.07.076 28783576

[B44] Jay T. M. Rocher C. Hotte M. Naudon L. Gurden H. Spedding M. (2004). Plasticity at hippocampal to prefrontal cortex synapses is impaired by loss of dopamine and stress: importance for psychiatric diseases. *Neurotox. Res.* 6 233–244. 15325962 10.1007/BF03033225

[B46] Juruena M. F. (2014). Early-life stress and HPA axis trigger recurrent adulthood depression. *Epilepsy Behav.* 38 148–159. 10.1016/j.yebeh.2013.10.020 24269030

[B47] Kalia L. V. Lang A. E. (2015). Parkinson’s disease. *Lancet* 386 896–912. 10.1016/S0140-6736(14)61393-3 25904081

[B48] Kiliaan A. J. Arnoldussen I. A. Gustafson D. R. (2014). Adipokines: a link between obesity and dementia. *Lancet Neurol.* 13 913–923. 10.1016/S1474-4422(14)70085-7 25142458 PMC4228955

[B49] Kim H. G. Jin S. W. Kim Y. A. Khanal T. Lee G. H. Kim S. J. (2017). Leptin induces CREB-dependent aromatase activation through COX-2 expression in breast cancer cells. *Food Chem. Toxicol.* 106 232–241. 10.1016/j.fct.2017.05.058 28571770

[B50] Komori T. Morikawa Y. Nanjo K. Senba E. (2006). Induction of brain-derived neurotrophic factor by leptin in the ventromedial hypothalamus. *Neuroscience* 139 1107–1115. 10.1016/j.neuroscience.2005.12.066 16564638

[B51] Kormos V. Gaszner B. (2013). Role of neuropeptides in anxiety, stress, and depression: from animals to humans. *Neuropeptides* 47 401–419. 10.1016/j.npep.2013.10.014 24210138

[B52] Kurosawa N. Shimizu K. Seki K. (2016). The development of depression-like behavior is consolidated by IL-6-induced activation of locus coeruleus neurons and IL-1β-induced elevated leptin levels in mice. *Psychopharmacology* 233 1725–1737. 10.1007/s00213-015-4084-x 26385227

[B53] Lee J. Y. Lim O. K. Lee J. K. Park Y. Kim C. Yoon J. W. (2015). The Association between serum leptin levels and post-stroke depression: a retrospective clinical study. *Ann. Rehabil. Med.* 39 786–792. 10.5535/arm.2015.39.5.786 26605177 PMC4654086

[B55] Liao G. Y. An J. J. Gharami K. Waterhouse E. G. Vanevski F. Jones K. R. (2012). Dendritically targeted Bdnf mRNA is essential for energy balance and response to leptin. *Nat. Med.* 18 564–571. 10.1038/nm.2687 22426422 PMC3327556

[B56] Liu B. B. Luo L. Liu X. L. Geng D. Li C. F. Chen S. M. (2015). Essential oil of Syzygium aromaticum reverses the deficits of stress-induced behaviors and hippocampal p-ERK/p-CREB/brain-derived neurotrophic factor expression. *Planta Med.* 81 185–192. 10.1055/s-0034-1396150 25590367

[B57] Liu Z. Zhang Y. Liu J. Yin F. (2017). Geniposide attenuates the level of Aβ1-42 via enhancing leptin signaling in cellular and APP/PS1 transgenic mice. *Arch. Pharm. Res.* 40 571–578. 10.1007/s12272-016-0875-9 28160136

[B58] Lu X. Y. Kim C. S. Frazer A. Zhang W. (2006). Leptin: a potential novel antidepressant. *Proc. Natl. Acad. Sci. U.S.A.* 103 1593–1598. 10.1073/pnas.0508901103 16423896 PMC1360555

[B59] Maayan L. Correll C. U. (2010). Management of antipsychotic-related weight gain. *Expert Rev. Neurother.* 10 1175–1200. 10.1586/ern.10.85 20586697 PMC3501406

[B60] Mangialasche F. Solomon A. Winblad B. Mecocci P. Kivipelto M. (2010). Alzheimer’s disease: clinical trials and drug development. *Lancet Neurol.* 9 702–716. 10.1016/S1474-4422(10)70119-8 20610346

[B61] Milaneschi Y. Lamers F. Bot M. Drent M. L. Penninx B. W. (2017). Leptin dysregulation is specifically associated with major depression with atypical features: evidence for a mechanism connecting obesity and depression. *Biol. Psychiatry* 81 807–814. 10.1016/j.biopsych.2015.10.023 26742925

[B62] Morris M. C. Compas B. E. Garber J. (2012). Relations among posttraumatic stress disorder, comorbid major depression, and HPA function: a systematic review and meta-analysis. *Clin. Psychol. Rev.* 32 301–315. 10.1016/j.cpr.2012.02.002 22459791 PMC3340453

[B63] Mulkey R. M. Malenka R. C. (1992). Mechanisms underlying induction of homosynaptic long-term depression in area CA1 of the hippocampus. *Neuron* 9 967–975. 1419003 10.1016/0896-6273(92)90248-c

[B64] Napoli I. Neumann H. (2009). Microglial clearance function in health and disease. *Neuroscience* 158 1030–1038. 10.1016/j.neuroscience.2008.06.046 18644426

[B65] Neher J. J. Neniskyte U. Zhao J. W. Bal-Price A. Tolkovsky A. M. Brown G. C. (2011). Inhibition of microglial phagocytosis is sufficient to prevent inflammatory neuronal death. *J. Immunol.* 186 4973–4983. 10.4049/jimmunol.1003600 21402900

[B66] Novkovic T. Mittmann T. Manahan-Vaughan D. (2015). BDNF contributes to the facilitation of hippocampal synaptic plasticity and learning enabled by environmental enrichment. *Hippocampus* 25 1–15. 10.1002/hipo.22342 25112659

[B67] Oania R. McEvoy L. K. (2015). Plasma leptin levels are not predictive of dementia in patients with mild cognitive impairment. *Age Ageing* 44 53–58. 10.1093/ageing/afu160 25349150 PMC4255616

[B68] Ottaway N. Mahbod P. Rivero B. Norman L. A. Gertler A. D’Alessio D. A. (2015). Diet-induced obese mice retain endogenous leptin action. *Cell Metab.* 21 877–882. 10.1016/j.cmet.2015.04.015 25980347 PMC4456263

[B69] Ozsoy S. Besirli A. Unal D. Abdulrezzak U. Orhan O. (2015). The association between depression, weight loss and leptin/ghrelin levels in male patients with head and neck cancer undergoing radiotherapy. *Gen. Hosp. Psychiatry* 37 31–35. 10.1016/j.genhosppsych.2014.09.002 25440723

[B70] Pan H. Guo J. Su Z. (2014). Advances in understanding the interrelations between leptin resistance and obesity. *Physiol. Behav.* 130 157–169. 10.1016/j.physbeh.2014.04.003 24726399

[B71] Park H. Ahn S. H. Jung Y. Yoon J. C. Choi Y. H. (2017). Leptin suppresses glutamate-induced apoptosis through regulation of ERK1/2 signaling pathways in rat primary astrocytes. *Cell. Physiol. Biochem.* 44 2117–2128. 10.1159/000485950 29241183

[B72] Park S. W. Lee C. H. Cho H. Y. Seo M. K. Lee J. G. Lee B. J. (2013). Effects of antipsychotic drugs on the expression of synaptic proteins and dendritic outgrowth in hippocampal neuronal cultures. *Synapse* 67 224–234. 10.1002/syn.21634 23335099

[B73] Pérez-González R. Alvira-Botero M. X. Robayo O. Antequera D. Garzón M. Martín-Moreno A. M. (2014). Leptin gene therapy attenuates neuronal damages evoked by amyloid-β and rescues memory deficits in APP/PS1 mice. *Gene Ther.* 21 298–308. 10.1038/gt.2013.85 24430238

[B74] Pérez-González R. Antequera D. Vargas T. Spuch C. Bolós M. Carro E. (2011). Leptin induces proliferation of neuronal progenitors and neuroprotection in a mouse model of Alzheimer’s disease. *J. Alzheimers Dis.* 24(Suppl. 2) 17–25. 10.3233/JAD-2011-102070 21335656

[B75] Plotsky P. M. Owens M. J. Nemeroff C. B. (1998). Psychoneuroendocrinology of depression. Hypothalamic-pituitary-adrenal axis. *Psychiatr. Clin. North Am.* 21 293–307. 9670227 10.1016/s0193-953x(05)70006-x

[B76] Procaccini C. Santopaolo M. Faicchia D. Colamatteo A. Formisano L. de Candia P. (2016). Role of metabolism in neurodegenerative disorders. *Metab. Clin. Exp.* 65 1376–1390. 10.1016/j.metabol.2016.05.018 27506744

[B77] Rockenstein E. M. McConlogue L. Tan H. Power M. Masliah E. Mucke L. (1995). Levels and alternative splicing of amyloid beta protein precursor (APP) transcripts in brains of APP transgenic mice and humans with Alzheimer’s disease. *J. Biol. Chem.* 270 28257–28267. 7499323 10.1074/jbc.270.47.28257

[B78] Sahu S. Ganguly R. Raman P. (2016). Leptin augments recruitment of IRF-1 and CREB to thrombospondin-1 gene promoter in vascular smooth muscle cells in vitro. *Am. J. Physiol. Cell Physiol.* 311 C212–C224. 10.1152/ajpcell.00068.2016 27281481

[B79] Sanacora G. Mason G. F. Rothman D. L. Behar K. L. Hyder F. Petroff O. A. (1999). Reduced cortical gamma-aminobutyric acid levels in depressed patients determined by proton magnetic resonance spectroscopy. *Arch. Gen. Psychiatry* 56 1043–1047. 10565505 10.1001/archpsyc.56.11.1043

[B80] Spina M. B. Squinto S. P. Miller J. Lindsay R. M. Hyman C. (1992). Brain-derived neurotrophic factor protects dopamine neurons against 6-hydroxydopamine and N-methyl-4-phenylpyridinium ion toxicity: involvement of the glutathione system. *J. Neurochem.* 59 99–106. 1613515 10.1111/j.1471-4159.1992.tb08880.x

[B81] Spinedi E. Gaillard R. C. (1998). A regulatory loop between the hypothalamo-pituitary-adrenal (HPA) axis and circulating leptin: a physiological role of ACTH. *Endocrinology* 139 4016–4020. 10.1210/endo.139.9.6291 9724060

[B82] Su Y. Carey L. C. Rose J. C. Pulgar V. M. (2012). Leptin alters adrenal responsiveness by decreasing expression of ACTH-R, StAR, and P450c21 in hypoxemic fetal sheep. *Reprod. Sci.* 19 1075–1084. 10.1177/1933719112442246 22534336 PMC4052208

[B83] Szczesna M. Kirsz K. Misztal T. Zieba D. A. (2019). Downregulation of LRb in VMH/DMH during the second half of gestation and upregulation of SOCS-3 in ARC in late-pregnant ewes - Implications for leptin resistance. *Gen. Comp. Endocrinol.* 274 73–79. 10.1016/j.ygcen.2019.01.003 30611814

[B84] Szkudelski T. Dłużewicz K. Sadoch J. Szkudelska K. (2017). Effects of the activation of heme oxygenase-1 on hormonal and metabolic changes in rats fed a high-fat diet. *Biomed. Pharmacother.* 87 375–380. 10.1016/j.biopha.2016.12.060 28068626

[B85] Talton O. O. Pennington K. A. Pollock K. E. Bates K. Ma L. Ellersieck M. R. (2016). Maternal hyperleptinemia improves offspring insulin sensitivity in mice. *Endocrinology* 157 2636–2648. 10.1210/en.2016-1039 27145007

[B86] Tartaglia L. A. Dembski M. Weng X. Deng N. Culpepper J. Devos R. (1995). Identification and expression cloning of a leptin receptor, OB-R. *Cell* 83 1263–1271. 8548812 10.1016/0092-8674(95)90151-5

[B87] Teunissen C. E. van der Flier W. M. Scheltens P. Duits A. Wijnstok N. Nijpels G. (2015). Serum leptin is not altered nor related to cognitive decline in Alzheimer’s disease. *J. Alzheimers Dis.* 44 809–813. 10.3233/JAD-141503 25352450

[B88] Tong J. Q. Zhang J. Hao M. Yang J. Han Y. F. Liu X. J. (2015). Leptin attenuates the detrimental effects of β-amyloid on spatial memory and hippocampal later-phase long term potentiation in rats. *Horm. Behav.* 73 125–130. 10.1016/j.yhbeh.2015.06.013 26135065

[B89] Wang B. Chandrasekera P. C. Pippin J. J. (2014). Leptin- and leptin receptor-deficient rodent models: relevance for human type 2 diabetes. *Curr. Diabetes Rev.* 10 131–145. 24809394 10.2174/1573399810666140508121012 PMC4082168

[B90] Wang Y. Xu J. Liu Y. Li Z. Li X. (2018). TLR4-NF-κB signal involved in depressive-like behaviors and cytokine expression of frontal cortex and hippocampus in stressed C57BL/6 and ob/ob Mice. *Neural Plast.* 2018:7254016. 10.1155/2018/7254016 29765402 PMC5885403

[B91] Weng Z. Signore A. P. Gao Y. Wang S. Zhang F. Hastings T. (2007). Leptin protects against 6-hydroxydopamine-induced dopaminergic cell death via mitogen-activated protein kinase signaling. *J. Biol. Chem.* 282 34479–34491. 10.1074/jbc.M705426200 17895242

[B92] Wu L. Chen G. Liu W. Yang X. Gao J. Huang L. (2017). Intramuscular injection of exogenous leptin induces adiposity, glucose intolerance and fatty liver by repressing the JAK2-STAT3/PI3K pathway in a rat model. *Gen. Comp. Endocrinol.* 252 88–96. 10.1016/j.ygcen.2017.02.012 28242305

[B93] Xing Y. Liu J. Xu J. Yin L. Wang L. Li J. (2015). Association between plasma leptin and estrogen in female patients of amnestic mild cognitive impairment. *Dis. Mark.* 2015:450237. 10.1155/2015/450237 26693203 PMC4677007

[B94] Yamamoto N. Tanida M. Kasahara R. Sobue K. Suzuki K. (2014). Leptin inhibits amyloid β-protein fibrillogenesis by decreasing GM1 gangliosides on the neuronal cell surface through PI3K/Akt/mTOR pathway. *J. Neurochem.* 131 323–332. 10.1111/jnc.12828 25039425

[B95] Zhang Y. Liu C. Zhao Y. Zhang X. Li B. Cui R. (2015). The effects of calorie restriction in depression and potential mechanisms. *Curr. Neuropharmacol.* 13 536–542. 26412073 10.2174/1570159X13666150326003852 PMC4790398

[B96] Zhang Y. Proenca R. Maffei M. Barone M. Leopold L. Friedman J. M. (1994). Positional cloning of the mouse obese gene, and its human homologue. *Nature* 372 425–432. 7984236 10.1038/372425a0

[B97] Zhao Y. Chen L. B. Mao S. S. Min H. X. Cao J. (2018). Leptin resistance was involved in susceptibility to overweight in the striped hamster re-fed with high fat diet. *Sci. Rep.* 8:920. 10.1038/s41598-017-18158-4 29343842 PMC5772526

